# Surgery to Treat Symptomatic Mobile Cecum Syndrome Is Safe and Associated with Good Recovery Outcomes

**DOI:** 10.1155/2018/4718406

**Published:** 2018-02-08

**Authors:** Manuela Cesaretti, Manuela Trotta, Irene Leale, Giuseppe Antonio Minetti, Giuseppe Cittadini, Fabrizio Montecucco, Giovanni Bruno Camerini, Giacomo Borgonovo

**Affiliations:** ^1^Dipartimento di Scienze Chirurgiche e Diagnostiche Integrate (DISC), Università degli Studi di Genova, 8 Largo Benzi, 16132 Genoa, Italy; ^2^Department of Radiology, Ospedale Policlinico San Martino, 10 Largo Benzi, 16132 Genoa, Italy; ^3^Ospedale Policlinico San Martino, 10 Largo Benzi, 16132 Genoa, Italy; ^4^First Clinic of Internal Medicine, Department of Internal Medicine, University of Genoa, 6 Viale Benedetto XV, 16132 Genoa, Italy; ^5^Centre of Excellence for Biomedical Research (CEBR), University of Genoa, 9 Viale Benedetto XV, 16132 Genoa, Italy

## Abstract

The mobile cecum syndrome includes a spectrum of conditions. The cecal volvulus represents the acute form, with typical feature of a bowel obstruction that needs immediate operative treatment. On the other hand, a chronic form of mobile cecum syndrome which is the most common form reported a history of intermittent crampy abdominal pain, distension, and constipation. In this study, five patients came to our attention during the last ten years, presenting different symptoms due to a mobile cecum. All patients were investigated by several diagnostic techniques according to the specific clinical setting. All patients were found to have the cecum and ascending colon unattached to the posterior peritoneum. Surgery was the treatment of choice. In our experience, the best diagnostic technique was computed tomography scan, especially if performed in the Trendelenburg position. We also propose virtual colonoscopy as a good option for diagnosis (in patients with chronic syndrome) and follow-up after surgery. In conclusion, laparoscopic approach guaranteed a good result, with no symptoms of recurrence, in both acute and elective treatments. The diagnosis of mobile cecum needs a high index of suspicion and a targeted radiological investigation. Surgery, especially laparoscopic cecopexy and appendectomy, is the recommended treatment.

## 1. Introduction

Mobile cecum is anatomically defined as an anomalous position of the right colon, cecum, and terminal ileum due to the failure of the right colon mesentery to fuse with the posterior parietal peritoneum. Embryogenesis of bowel is a complex process that begins during the 5th gestational week and involves three phases: herniation, return to the abdomen, and fixation [1, 2]. Anomalies of rotation and fixation of the gastrointestinal tract are frequently associated with other embryological defects, but per se the suspension on a mesentery of the cecum and ascending colon may allow them to freely rotate and cause a wide spectrum of symptoms included in the “mobile cecum syndrome.” This condition can be asymptomatic, as it has been found in 11.2% of autopsies [3, 4]. On the other hand, it can also clinically manifest as a chronic syndrome including constipation, abdominal distension, and recurrent abdominal pain or, more rarely, as an acute bowel obstruction due to cecal volvulus (1–1.5% of all adult intestinal obstruction) [5]. This study reports a series of five patients presenting for a symptomatic mobile cecum that required surgical treatment (mostly by laparoscopy).

## 2. Methods

In the last ten years, five patients were admitted at the Surgery Unit of San Martino Hospital (Genoa, Italy), since they presented different symptoms due to an anomalous position of the cecum. Two patients came after a history of recurrent lower quadrant pain and abdominal distension (chronic form of the disease); the other three patients arrived with an acute form of cecal volvulus (acute form for the disease). Patients gave written informed consent prior to entering the study. The protocol adheres to the principles of the Declaration of Helsinki. The cases of acute and chronic forms of the diseases are summarized in Table 1. The grade of lack of peritoneal attachment of the right colon was graded as follows: I (cecum retroperitoneal or with little mobility), II (wide mobility, crossing the midline), and III (maximum mobility, reaching the left abdomen) [6].

Diagnosis was obtained by several diagnostic techniques (ultrasound, abdominal radiography, computed tomography, and virtual colonoscopy), according to the specific clinical setting.

All patients were examined using a computed tomography (CT) scanner (GE LightSpeed 16) with the following scan parameters: slice thickness of 1.2 mm, tube rotation time of 0.8 s, and KV/mAs of 120/Mod. The scan was performed with the patient supine from the diaphragmatic domes to the pubic symphysis before and after injection of intravenous nonionic, uroangiographic contrast material (Ultravist 370®; Bracco, Milan, Italy) into the cubital vein with an 18-gauge needle connected to an automatic injector. The parameters were as follows: volume of 1,5 ml/kb of body weight (maximum value: 120 mL) and an injection rate of 3 ml/s. Images were obtained during the arterial and portal phase, and the scan delay was determined automatically using the SmartPrep tracking technique. 3% diatrizoate meglumine (Gastrografin®; Bracco, Milan, Italy) was administered as positive rectal contrast material. To stress the spontaneous colic malposition, CT scans performed in Trendelenburg position were also acquired. The majority of patients were operated by laparoscopic surgery. In case of massive overdistended bowel loops, a laparotomy was chosen. Several surgical procedures were carried out according to the ischemic state of the bowel: in case of ischemic colon, a right colectomy was performed; otherwise, a cecopexy and appendectomy were performed. Laparoscopic cecopexy procedure is briefly summarized below. After the induction of general anesthesia, the patient was placed in the supine position. A 15 mm skin incision was made just below the umbilicus, a 12 mm trocar was inserted, and pneumoperitoneum was established by insufflation with carbon dioxide to 12 mmHg abdominal pressure. Two 5 mm trocars were inserted under laparoscopic guidance: one in the middle lower abdominal region and the other in the left lateral region. First step was the careful examination of the abdominal cavity, looking for evident intraperitoneal adhesions, macroscopic gastroenteric diseases, and the right colon position. Cecopexy was done by suturing the cecum and the ascending colon under a flap of parietal peritoneum expressly created with interrupted absorbable sutures.

## 3. Report of Cases

### 3.1. Acute Form of the Disease

*Case 1. *A 20-year-old man was admitted to the Emergency Department with a six-hour-long diffuse abdominal pain, vomiting, and constipation. His medical history did not present remarkable episodes, except for a chronic recurrence of intermittent lower right quadrant pain. No previous surgery was reported. At physical examination, he presented diffuse abdominal tenderness, more evident in upper left quadrant, with asymmetric meteorism and rebound tenderness. Digital rectal examination revealed no stools in rectal ampulla. Laboratory tests showed a white blood cell count of 13800 cells/mm^3^ and elevated C-Reactive Protein (CRP) (130 mg/L).

**Figure 1:**
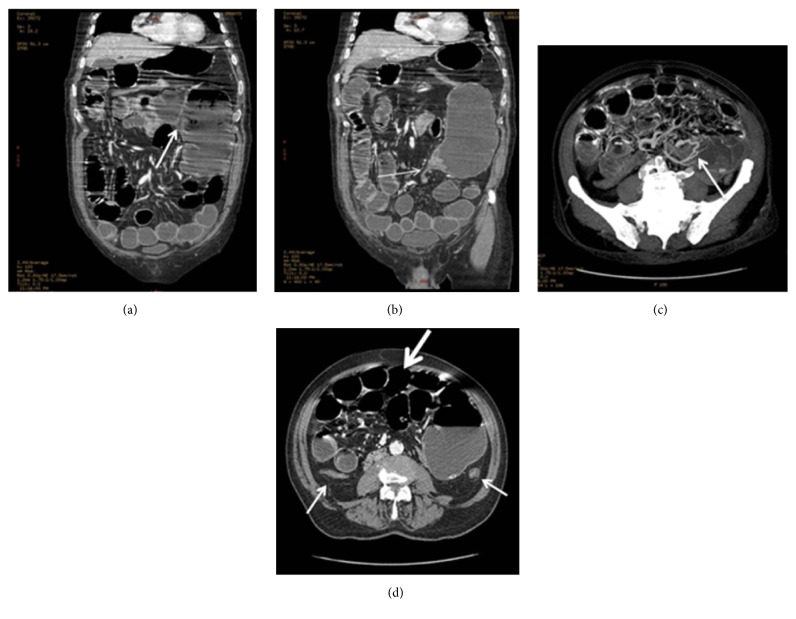
(a) Medially placed ileocecal valve (arrow). (b) Dilated, twisted cecum with tip pointing to left upper quadrant (arrow). (c) “Whirlpool” sign: tightly twisted mesentery and bowel vessels. (d) Dilated small bowel loops with air fluid levels (thick arrow) and distended gas or fluid-filled small bowel (white arrows).

Diagnosis of bowel obstruction was confirmed by an abdominal plain radiograph that revealed a grossly dilated bowel segment with haustra in the right abdomen. No free gas was detected. A CT scan demonstrated a dilated and twisted cecum with tip pointing to left upper quadrant, twisted mesentery, and small bowel vessels. Distal colon was collapsed. All these findings were suggestive of cecal volvulus (Figure 1).

Exploratory laparoscopy revealed a notable distension of the cecum, ileum, and jejunal loops. The cecum was rotated around its axis and located in the left abdominal quadrant. Distal colon beyond the rotation was collapsed. Free fluid was found in the abdomen and it was collected for microbiological culture, which resulted to be negative. After derotation of the right colon, the intestine recovered completely, so an appendectomy and cecopexy using a right gutter peritoneal flap were performed. Postoperative course was uneventful and the patient was discharged on the 5th postoperative day. After five years, he does not suffer from abdominal pain anymore.

**Figure 2:**
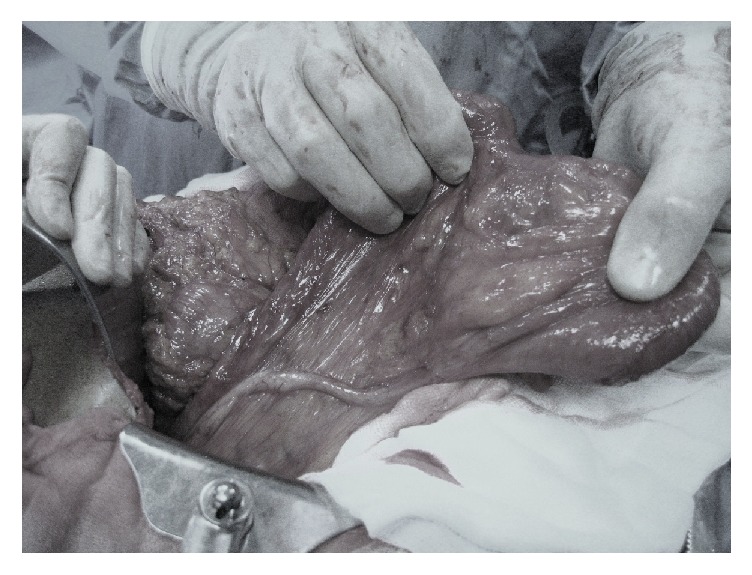
Elongation of the ileocolic pedicle without lateral peritoneal attachments and with hypermobile cecal apex, which could be directed toward the upper quadrant. A retrocecal, ascending appendix can also be noticed.

*Case 2. *An 82-year-old man presented with a two-day history of fever, acute diffuse abdominal pain, diarrhea, and then constipation. Neither vomiting nor fever was reported. Past medical history included acute prostatitis and arterial hypertension treated with amiloride chlorhydrate and hydrochlorothiazide. The patient also had a history of chronic abdominal pain and distension that was relieved by passing flatus. Physical examination revealed tenderness in the right lower quadrant. Rectal examination showed residual normal stool in rectal ampulla. Laboratories' studies showed no abnormalities except for leukocytosis (15000/mm^3^). Abdominal radiography showed air fluid levels of the small bowel. CT scan demonstrated ileal peritoneal adhesion with markedly dilated small bowel loops (>2.5 cm) and collapsed colon. A nasogastric tube was placed to decompress the bowel but did not result in improvement of patient's situation. Consequently, he underwent urgent laparotomy that revealed a volvulus of the cecum. The cecum had an elongation of the ileocolic pedicle without lateral peritoneal attachments and it was situated in the upper left abdominal quadrant (Figure 2). The bowel was ischemic but not perforated; nevertheless a right colectomy was performed. Histological exam of the resected specimen showed a thin and necrotic bowel. Postoperative course was uneventful and he was discharged on the 7th postoperative day. The patient is currently asymptomatic after two years of follow-up.

*Case 3. *A 40-year old woman presented with a three-day history of persistent diffuse acute abdominal pain and vomiting. Her medical problems included intermittent abdominal distension and constipation for several years. An appendectomy was performed twenty years previously. No medication was reported. The abdomen was symmetrically distended with tenderness, but there was neither muscle rigidity nor Blumberg's sign. Laboratory studies revealed leukocytosis (12000/mm^3^). Plain abdominal X-ray showed markedly dilated loops with air fluid levels. Transverse colon appeared collapsed. These findings suggested the presence of a bowel obstruction in the ascending colon. A CT scan confirmed it and aroused the suspicion of a volvulus of the right colon. The patient underwent urgent laparotomy that revealed dilated small bowel loops and a volvulus of the cecum located in the central abdominal quadrant. The cecum was derotated and a fixation of the right colon to the right parietocolic peritoneal space using a peritoneal flap was performed. On the 3rd day after surgery, the patient had fever due to a pneumonia treated with quinolone. She was discharged on the 15th postoperative day. She remains asymptomatic ten years after the cecopexy.

### 3.2. Chronic Form of the Disease

**Figure 3:**
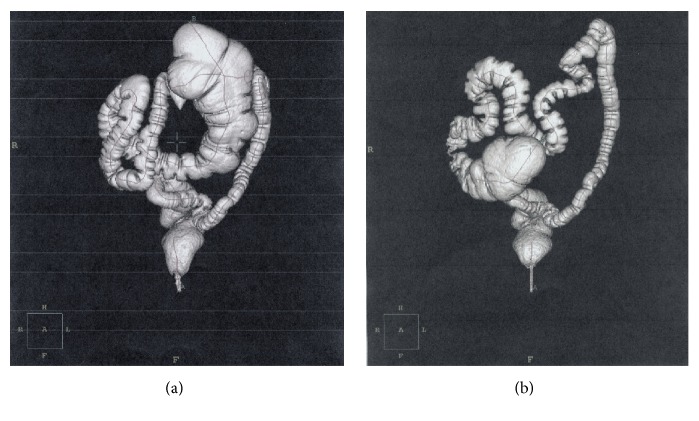
(a) Virtual colonoscopy. Three-dimensional volume-rendered CT colonography (anteroposterior view) showed an elongated right colon with the cecum dislocated in the upper left quadrant. (b) Virtual colonoscopy one year after surgery. Three-dimensional volume-rendered CT colonography (anteroposterior view) demonstrated the cecum and the ascending colon in the physiological position.

*Case 4. *A 57-year-old woman arrived with a five-month history of intermittent colicky periumbilical pain, constipation, and abdominal distention. Past medical history included colpohysterectomy for uterine fibroids, hiatal hernia treated conservatively, and arterial hypertension treated by atenolol. At physical examination, the patient's abdomen was distended with diffuse tenderness, without muscle guarding. The bowel sounds were normal. Laboratory blood tests were normal. During a previous emergency admission for a severe attack of abdominal pain, ultrasound and plain abdominal radiographs were performed. They showed dilated bowel and a medial position of the appendix. After one month, a virtual colonoscopy was performed and demonstrated a large right colon in the left abdominal quadrant (Figure 3(a)), so a mobile cecum syndrome was diagnosed.

The patient underwent a laparoscopy that revealed a long and mobile right colon, without attachments to the posterior abdominal wall. No other abnormalities were found. A cecopexy using a lateral peritoneal flap was performed. A complementary appendectomy was carried out as well. Postoperative course was uneventful and the patient was discharged on the 3rd postoperative day. She has not had pain for one year and another virtual colonoscopy demonstrated the physiological position of the cecum (Figure 3(b)).

*Case 5. *A 54-year-old woman was admitted to our hospital with a six-month history of crampy diffuse abdominal pain and constipation. Ten years previously, she underwent a laparoscopic ovariectomy for endometriosis. At physical examination, the patient's abdomen appeared diffusely tender, without signs of peritonitis. Laboratory tests were negative. Ultrasound and plain abdominal radiographs taken at another hospital showed dilated bowel loops throughout the abdomen. Rectal contrast CT examination performed a week before surgery showed an enlarged right colon in the left abdominal quadrant. A mobile cecum syndrome was diagnosed. The patient underwent laparoscopic surgery that revealed a malposition of a mobile right colon, unattached to the posterior peritoneal abdominal wall, without signs of ischemia. Appendectomy and cecopexy were performed. The patient recovered after the operation and was discharged on the 3rd postoperative day. Four years after the surgery, no further recurrence of abdominal pain was reported.

## 4. Discussion

Symptomatic presentation of an anomalous cecum fixation includes recurrent abdominal pain and constipation due to the intermittent distension and sufferance of the cecum or acute bowel obstruction due to cecal volvulus. The presence of the recurrent pattern, known as mobile cecum syndrome, is reported in about 50% of the patients before the onset of acute volvulus [4]. Symptoms of the chronic syndrome are relieved after passing flatus or using laxative therapy. During symptomatic episodes, physical examination reveals abdominal tenderness and high pitched bowel sounds. Laboratory tests are negative. When there are two concomitant requisites, such as hypermobile cecum and a point of fixation with torsion, a cecal volvulus may occur. Clinically, it is an acute bowel obstruction, which has various features depending on the severity and the duration of the intestinal obstruction, ranging from the subacute obstruction to the acute fulminant pattern [5]. Symptoms are not specific, with different intensity, but the most common presentations are acute abdominal pain (80%), distension (80%), constipation (60%), and vomiting (28%) [7].

While mobile cecum syndrome could be suitable to elective surgery, cecal volvulus, if not rapidly treated, may evolve to intestinal strangulation with bowel ischemia, gangrene, and perforation. Because of this serious clinical condition, it is very important to make a prompt diagnosis. Since clinical and laboratory tests are often not specific, for both mobile cecum syndrome and acute presentation, radiological investigations play a central role in diagnosis. Patients with mobile cecum syndrome often have extensive work-ups before a correct diagnosis. Ultrasonography is always the first examination to be carried out because of its cost-effectiveness. It allows excluding appendicitis or macroscopic lesions of the intestine. Sometimes it can detect anomalies of right colon position but its findings are generally nonspecific [8]. CT scan (with endoluminal contrast in nonacute patients) can reveal medialization of appendix and a suspicion of cecum malposition. Scan performed in Trendelenburg position can show a hypermobile cecum with cecal apex directed toward the left upper quadrant. This sign is highly specific for mobile cecum. Virtual colonoscopy can be also a valid technique for both diagnosis (in patients affected by mobile cecum syndrome) and follow-up after the cecopexy. When acute bowel obstruction is suspected, radiological investigations become crucial to demonstrate the level and the cause of obstruction.

Cecal volvulus is mostly diagnosed on abdominal plain radiographs and CT images. In a large series, however, abdominal X-ray is rarely diagnostic, and therefore CT scan is usually performed [9]. Barium enema can also be used, with an accuracy of 88% [4, 9]. This technique can show a collapsed colon downstream the pivot point and if the obstruction is incomplete, barium may trickle past the site of obstruction and the twist could be visualized in detail. Because of the risk of overdistension and perforation of the cecum during this exam, it is not indicated in emergency. Additional drawbacks are signs of peritonitis, rectal bleeding, radiographic sign of gas in the bowel wall, and pneumoperitoneum [10]. Literature reports cases of occasional successful cecal volvulus reduction after barium enema administration [11]. Severe cecal distension, cecal apex in left upper quadrant, collapsed distal colon, small bowel distension, whirlpool sign (the superior mesenteric artery wrapped by coils of superior mesentery vein and bowel), ileocecal twist, and transition points are the most common CT findings [12]. Differential diagnosis includes gastric dilation, sigmoid volvulus, small bowel volvulus, and colon obstruction with a competent ileocecal valve [7]. After the achievement of a correct diagnosis, a tailored treatment should be performed. Mobile cecum syndrome should be treated surgically, due to the recurrence of abdominal pain and the risk of cecal volvulus.

Authors suggest associating appendectomy with cecopexy in order to increase the fixation to parietal peritoneum due to postappendectomy adhesions [13]. Literature reports recurrence of syndrome after simple cecopexy, but most of these procedures use only simply suturing the cecum to lateral peritoneum [3, 4]. Rabinovici and coworkers [9] reported that cecopexy has a recurrence rate of 13%. In case of cecal volvulus, endoscopy has been proposed as initial treatment. However, it is usually associated with unsuccessful reduction and high risk of perforation, and therefore it is considered as a treatment of limited interest [14, 15]. Surgery offers different options of surgical treatment, including detorsion, cecopexy (with or without appendectomy), colectomy, or cecostomy. Treatment should be tailored and based on patient's performance status, viability of the bowel, risk of recurrence, and surgeon's skill in laparoscopic procedure. There is generally an agreement on the fact that, in case of gangrene and perforation of the bowel, it should be resected. Colectomy of course eliminates the possibility of volvulus recurrence, but it is associated with higher morbidity and mortality [16]. Improvements in surgical technique and perioperative cares had led to reduction of the mortality and morbidity rate. Cecostomy should be avoided because it results in a high rate of complications. In case of early diagnosis of cecal volvulus and in the absence of a massive loops distension (>2.5 cm), a laparoscopic approach should be attempted.

Literature reports only eight cases of mobile cecum syndrome or cecal volvulus treated by laparoscopic surgery [3, 6, 17–21]. In our experience, laparoscopic cecopexy using a peritoneal flap is a valid therapeutic approach that could be adopted in both chronic and acute manifestations of anomalous cecum position with excellent results and no recurrence of symptoms in a medium term follow-up.

## 5. Conclusion

Anomalous position of the right colon is quite common, but it is rarely symptomatic. Indeed diagnosis of a clinically evident mobile cecum needs a high index of suspicion. CT scan is the best imaging technique available for a correct diagnosis, especially in Trendelenburg position. Surgery is the treatment of choice in order to prevent or solve volvulus of the cecum and recurrence of disease. In this report, the most performed procedure is laparoscopic cecopexy with peritoneal flap associated with appendectomy. Only one patient presented signs of bowel ischemia and a right colectomy became necessary. Laparoscopic suturing of the cecum can be performed in the same way as in open surgery but with better functional results. In our experience, recurrences were absent in a medium term follow-up.

## Figures and Tables

**Table 1:** Summary of clinical cases' characteristics.

Clinical case	Patient age (years)/sex (F/M)	Laboratory test alteration	CT scan/virtual colonoscopy positivity	Grade of lack of peritoneal attachment of the right colon (I-II-III)	Surgical treatment	Postoperative day of discharge
*Acute form*						
Case 1	20/M	Yes	Yes	II	Laparoscopy	5th day
Case 2	82/M	Yes	Yes	II	Laparotomy	7th day
Case 3	40/F	Yes	Yes	II	Laparotomy	15th day

*Chronic form*						
Case 4	57/F	No	Yes	II	Laparoscopy	3rd day
Case 5	54/F	No	Yes	II	Laparoscopy	3rd day
